# Integrative Analysis of DNA Methylation and Gene Expression Identified Follicular Thyroid Cancer-Specific Diagnostic Biomarkers

**DOI:** 10.3389/fendo.2021.736068

**Published:** 2022-03-14

**Authors:** Yunjin Yao, Peiwei Xu, Tianxing Ying, Yue Wang, Xumeng Wang, Liqi Shu, Zhe Mo, Zhijian Chen, Xiaofeng Wang, Weibin Wang, Lisong Teng, Xiaoming Lou

**Affiliations:** ^1^ Department of Surgical Oncology, The First Affiliated Hospital, College of Medicine, Zhejiang University, Hangzhou, China; ^2^ Zhejiang Provincial Center for Disease Control and Prevention, Hangzhou, China; ^3^ Warren Alpert School of Medicine, Brown University, Providence, RI, United States

**Keywords:** FTC, DNA methylation, diagnostic biomarker, gene expression profile (GEP), integrative “omics”

## Abstract

The diagnosis of follicular thyroid carcinoma (FTC) prior surgical resection remains a challenge, as routine screening methods, such as ultrasound or even FNAB, could not diagnose FTC preoperatively. Here, we performed an integrative analysis of DNA methylation and RNA array data from our own cohort (14 Follicular thyroid carcinoma vs 16 Benign thyroid lesion) to identify thyroid cancer-specific DNA methylation markers. We first identified differentially methylated and expressed genes and examined their correlations. Candidate DNA methylation sites were selected and further verified in validation set. Among all candidate methylation sites, cg06928209 was the most promising site as a molecular marker for early diagnosis, with a sensitivity of 90%, a specificity of 80% and an AUC of 0.77. Overall, our study demonstrates the potential use of methylation markers in FTC diagnosis and may boost the development of new epigenetic therapies.

## Introduction

Follicular cell‐derived thyroid cancer is the most common endocrine malignancy. About 90-95% of thyroid cancers are follicular cell‐derived and can be classified into differentiated thyroid cancers, including papillary thyroid carcinoma (PTC) and follicular thyroid carcinoma (FTC), poorly differentiated thyroid cancers and anaplastic thyroid cancers (1). Approximately 5% of clinically palpable thyroid nodules are thyroid cancers (2). Currently, fine needle aspiration biopsy (FNAB) is the gold standard for screening and diagnosis of thyroid nodules (3). Despite its high accuracy, approximately 17% of FNAB results are classified as indeterminate cytology (4). Subsequently, thyroidectomy is usually recommended while more than 60% of these resected nodules are ultimately diagnosed as non-malignant (4, 5), which means that more than half of those patients underwent unnecessary surgery and may require lifelong thyroid hormone replacement (6, 7).

Therefore, accurate preoperative diagnosis is important to improve the quality of patients’ life, yet the diagnosis of FTC has been a challenge, which requires postoperative pathologic evaluation (4). Routine screening methods such as Ultrasound and FNAB have difficulties to distinguish FTC from benign thyroid nodules, especially follicular adenoma (2–4). Thus, there is an urgent need to advance the preoperative diagnostic tools for FTC.

Several molecular tests have been proposed to improve the accuracy of indeterminate FNAB cytology and avoid unnecessary or potentially harmful surgery for these patients. The investigation for genomic aberrations in FNAB, including BRAF and RAS point mutations, and PAX8/PPARγ and RET/PTC rearrangements have been explored (8). Recently, rare alterations described by next generation sequencing have also been reported to be diagnostically informative. Nevertheless, in many tumors these alterations are not observed, resulting in an elevated number of false‐negative results (9–11). In the current era of molecular diagnostics, advanced methods are expected to continue to improve the diagnosis of thyroid nodules.

DNA methylation is considered to be a promising molecular marker for clinical testing, as DNA is more stable than RNA and proteins (12–14). Several studies have reported that some genes exhibit differential methylation in thyroid cancer, suggesting that these alterations may be useful in differentiating benign and malignant thyroid nodules (15–18). Based on the comparison of DNA methylation sites and RNA expression profiles between FTC and benign thyroid lesion (BTL) samples, our study intends to identify DNA methylation sites and their corresponding genes that are of greater clinical significance through the integrated analysis of the two results. Then potential molecular markers are identified to distinguish FTC and BTL.

## Materials and Methods

### Study Cohort and Design

**Figure 1:**
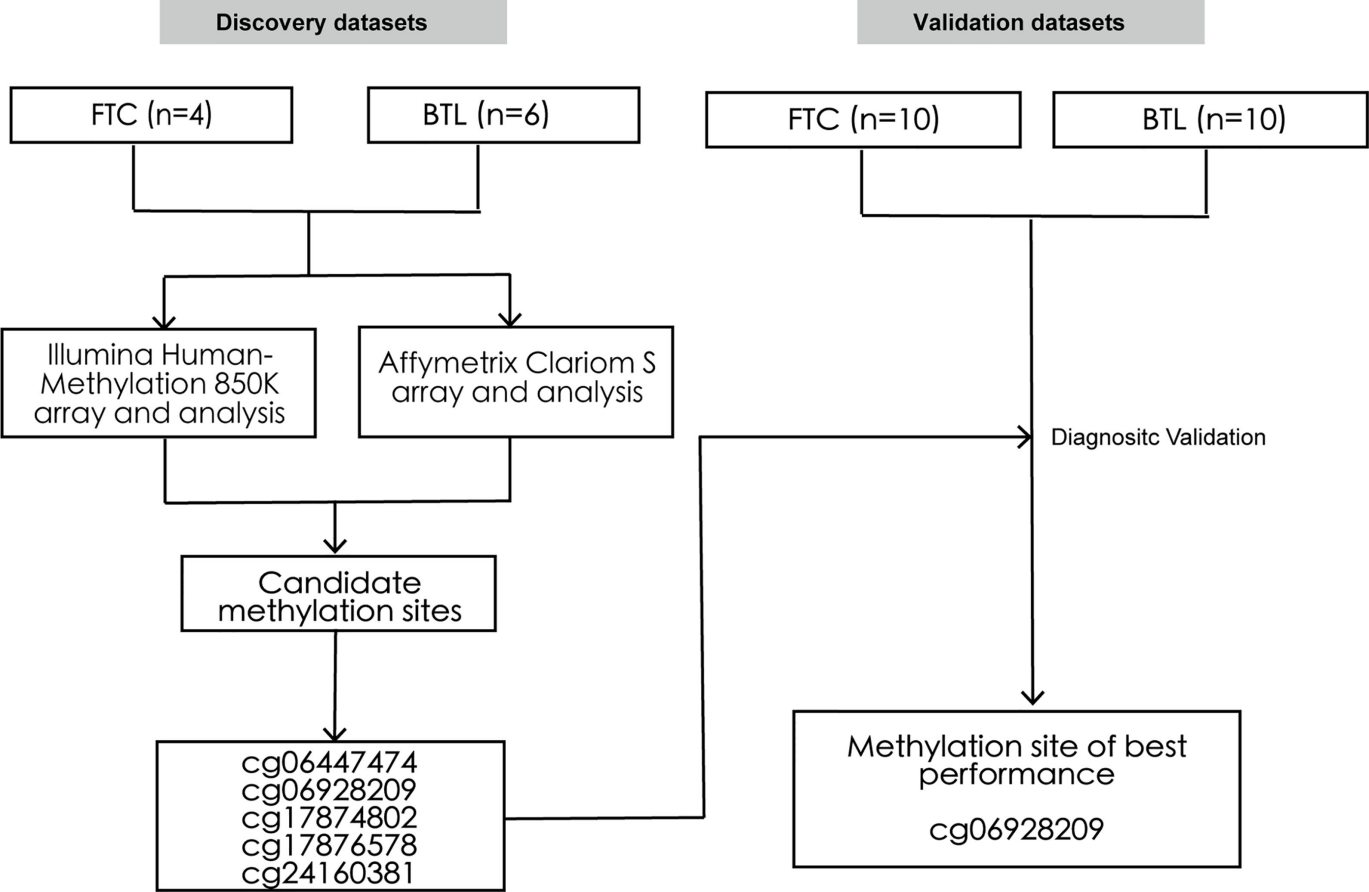
Study design. The schematic diagram represents the strategy for discovery and validation of the FTC-specific methylated sites for preoperative diagnosis. FTC, Follicular thyroid carcinoma; BTL, Benign thyroid lesion.

Thirty patients who underwent surgical resection of thyroid nodules in our medical center between 2016 to 2019 were randomly chosen in this study. All tissue samples were retrieved from formalin-fixed paraffin-embedded blocks and reviewed by an experienced attending pathologist. Among these samples, 10 were used as the discovery set, including 4 FTC and 6 BTL. And the rest were used as the external validation set, including 10 FTC and 10 BTL. Then, the DNA methylation profiles and RNA expression profiles of the samples in the discovery set were obtained by Illumina HumanMethylation850K array and Affymetrix Clariom S array respectively. Then, differential methylation and expression analyses were conducted, which were followed by integrative analysis of methylation sites and genes. Then, five methylation sites were identified and validated by Pyrosequencing in validation dataset. **Figure 1** summarized the design of this study. The Institutional Ethics Committee approved the study. All thirty patients were advised and research informed consents were obtained.

### RNA Differential Expression Analysis

Total RNA was extracted and purified using RecoverAllTM Total Nucleic Acid Isolation (Cat#AM1975, Ambion, Austin, TX, US) following the manufacturer’s instructions. Then, the total RNA was amplified, labeled and purified by Affymetrix WT PLUS Reagent Kit (Cat#902280, Affymetrix, Santa Clara, CA, US), Ovation FFPE WTA System (Cat#3403, NuGEN, San Carlos, CA, US) and FL-Ovation™ cDNA Biotin Module V2 (Cat#4200, NuGEN, San Carlos, CA, US). Array hybridization and washing was performed using GeneChip^®^ Hybridization, Wash and Stain Kit (Cat#900720, Affymetrix, Santa Clara, CA, US) in a Hybridization Oven 645 (Cat#00-0331-220V, Affymetrix, Santa Clara, CA, US) and a Fluidics Station 450 (Cat#00-0079, Affymetrix, Santa Clara, CA, US). Arrays were scanned by Affymetrix GeneChip^®^ Scanner 3000 (Cat#00-00213, Affymetrix, Santa Clara, CA, US) to obtain raw data. The raw data were normalized by Expression Console software of Affymetrix company. After normalization, the fold changes and p-values of different genes were calculated by the formula: foldchange = average (power (2, signal (FTC)))/average (power (2, signal (BTL))) and t-test separately. Differentially expressed genes (DEGs) were reported if the fold change was >2 or <0.5 and the p-value was smaller than 0.05. At last, Unsupervised consensus clustering was performed by R package “pheatmap” to obtain the heatmap of DEGs.

### DNA Differential Methylation Analysis

DNA was isolated from the samples using OMEGA TISSUE DNA Kit according to the manufacturer’s instructions. DNA (500 ng) was treated with bisulfate using an EZ DNA Methylation Gold Kit (Zymo Research, Irvine, CA), according to the manufacturer’s instructions. The methylation of DNA was assayed on the Infinium MethylationEPIC BeadChip (Illumina, San Diego, CA) using the Illumina HD methylation assay kit from Shanghai Biotechnology Corporation.

DNA methylation data were analyzed using the methylation analysis module within BeadStudio software employing default parameters (Illumina, Inc., San Diego CA, USA). The raw intensity data were loaded to a biocondutor package “minfi” (verison 1.25.1). The raw data were normalized using the subset-quantile within array normalization (SWAN) method and probes with a detection p-value over 0.01 in at least one sample were excluded from further analysis. Methylation values, referred to asβ-values, were calculated as the ratio of the methylated signal intensity to the sum of the methylated and unmethylated signals after background subtraction. The β-values were reported as a DNA methylation score ranging from 0 (completely unmethylated) to 1 (fully methylated). Differentially methylated sites were selected in IMA (version 3.1.2). In this study, we assessed the mean-difference β-value (Δβ) between the two sample groups for each site. Specifically, we considered a probe as differentially methylated if the absolute Δβ was higher than 0.1 and the statistical test was significant (p value <0.05).

### Combination of Methylation Profiles and Expression Profile

On the basis of both expression profiling and DNA methylation differential analysis, DMSs were annotated to specific genes by annotation table from Illumina website (downloaded from https://webdata.illumina.com/downloads/productfiles/methylationEPIC/infinium-methylationepic-v-1-0-b5-manifest-file-bpm.zip), R package “ChAMP” and annotation information downloaded from UCSC Xena. Then we used function “merge” to match the DMSs to genes they located on. Specific locations of DMSs were also taken into consideration, where DMSs around the transcription start site (TSS) were more preferred. Since it is reported that DNA methylation is associated with gene silencing (19), DMSs with an inverse relationship between expression and methylation were of more interest. Genes’ information and background were also taken into consideration to select genes were interested in. Overall, five DMSs with a SNP distance over 10 bps and relatively large absolute Δβ were selected as candidate methylation sites.

### Bisulfite‐Pyrosequencing Analysis

The DNA methylation pattern of five selected DMSs was evaluated by bisulfite‐pyrosequencing in the validation set. PCR and sequencing primers were designed to evaluate the same DMSs identified by microarray analysis. After PCR amplification, a pyrosequencing reaction was carried out using Pyromark Q96 reagents (Qiagen). The candidate methylation sites were evaluated in the validation set.

### Statistical Analysis

Unsupervised consensus clustering was conducted using default method of argument “clustering_method”. Function “testfunc” in IMA package was used to determine p value. The PCA analysis was performed by R function “prcomp” and ROC analysis was performed by the “pROC” package to determine the area under the curve (AUC). All data analyses were performed with R (R version 3.6.1).

## Results

### Basic Clinical Information of the Cohort

This cohort included 12 males (40.0%) and 18 females (60.0%). The age distribution of the patients at the time of diagnosis ranged from 14 to 79 years, with a median of 52 years and a mean of 47.50 ± 16.12 years. As shown in **Supplementary Table 1**, all the FTC patients had no lymphatic metastases or distant metastases and only 2 of them were in stage II according to the 8^th^ AJCC TNM staging system.

### Differentially Methylated Sites in FTC and BTL

**Figure 2:**
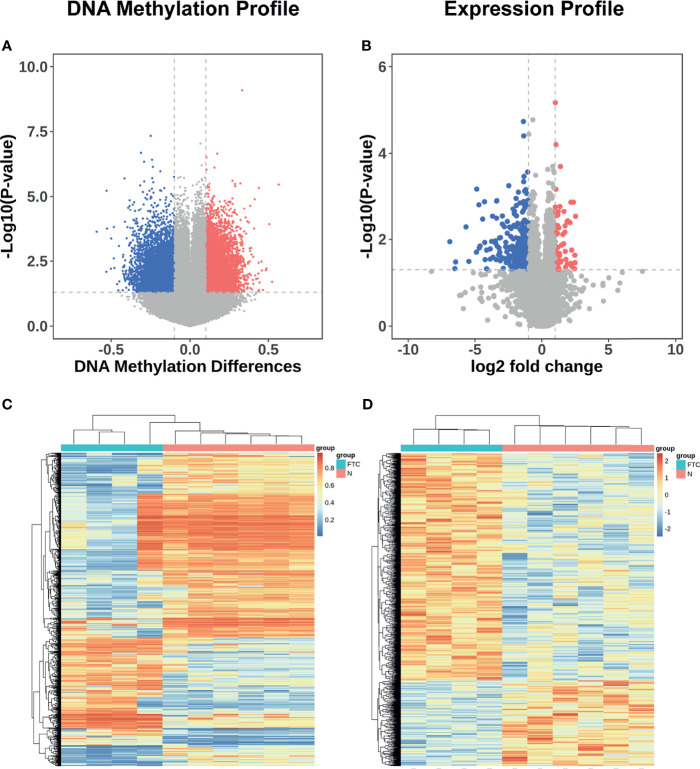
DNA methylation and mRNA expression profile of the discovery set. **(A)** Volcano plots showing differentially methylated sites in discovery datasets. Sites with △β value > 0.1 and P value < 0.05 were defined as significantly hypermethylated methylation sites, which were showed in red; meanwhile, those with adjusted P value < 0.05 and △β value < -0.1 were defined as significantly hypomethylated methylation sites, which were showed in blue. The other miRNAs were showed in grey. **(B)** Volcano plots showing differentially expressed mRNAs in discovery datasets. mRNAs with adjusted P value < 0.05 and log2FC > 1 were defined as significantly overexpressed mRNAs, which were showed in red; meanwhile, those with adjusted P value < 0.05 and log2FC < -1 were defined as significantly under-expressed mRNAs, which were showed in blue. The other mRNAs were showed in grey. **(C)** Heatmap showing a promising result of the hierarchical clustering analysis using differentially methylated sites to distinguish different samples in discovery dataset. **(D)** Heatmap showing a promising result of the hierarchical clustering analysis using differentially expressed genes to distinguish different samples in discovery dataset.

Of all 865,267 methylation sites compared between FTC and BTL, 13155 were differentially methylated. Among the differentially methylated sites, 6902 were hypermethylated in FTC and 6253 were hypomethylated in FTC. All the differentially methylated sites were displayed in volcano plot (**Figure 2A**). And a thousand of Sites with highest |△β| were included in heat-map (**Figure 2C**), which shows the samples were clearly separated into two groups in unsupervised consensus clustering. It means these Sites was promising in discriminating FTC from BTL.

### Differentially Expressed Genes in FTC and BTL

Similarly, 31824 genes were compared between FTC and BTL, while 346 genes are differentially expressed, in which, 77 were over expressed in FTC and 269 were under expressed in FTC. All the differentially expressed genes were displayed in volcano plot (**Figure 2B**). All the differentially expressed genes were included in heatmap (**Figure 2D**), which shows the samples were also clearly separated into two groups in unsupervised consensus clustering.

### Combination of Methylation Profiles and Expression Profile

**Figure 3:**
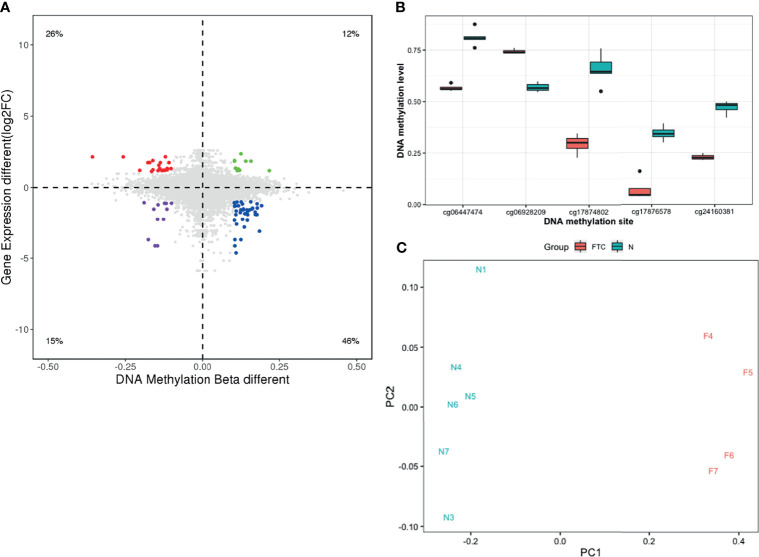
**(A)** The result of correlation analysis showing differentially methylated sites were significant between FTC and BTL while inversely correlated with gene expressions. **(B)** Box plot showing significant different methylation levels of the 5 selected methylated sites in FTC patients compared with the BTL patients in discovery dataset. **(C)** Primary component analysis (PCA) shows FTC and BTL samples can be separated into two groups correctly when using the 5 selected methylation sites.

To identify DNA methylation changes with concomitant changes in gene expression, we integrated the gene expression profiles and DNA methylation profiles of the FTC and BTL. Differentially methylated sites between the two groups were identified and combined with data from gene expression profiles. A total of 80 differentially methylated sites were significant between FTC and BTL and 58 were inversely correlated with gene expressions. Both DNA methylation profiles and mRNA expression profiles based on unsupervised hierarchical clustering identified two unique clusters that had distinct signatures (**Figures 2C, D**). **Figure 3A** shows the result of integrated analysis of DNA methylation and mRNA expression in FTC *vs.* BTL.

### Candidate Methylation Sites

According to the methods, 5 methylation sites were selected as candidate methylation sites. The methylation level of theses candidate methylation sites between FTC and BTL were displayed in **Figure 3B** as a boxplot, which shows significant differences between the two groups. In addition, **Figure 3C** shows the result of the principal component analysis (PCA). The samples gathered together within the same group and stayed away from the samples of different group. That means the candidate methylation sites performed well in distinguishing FTC from BTL.

### External Validation of Candidate Methylation Sites

**Figure 4:**
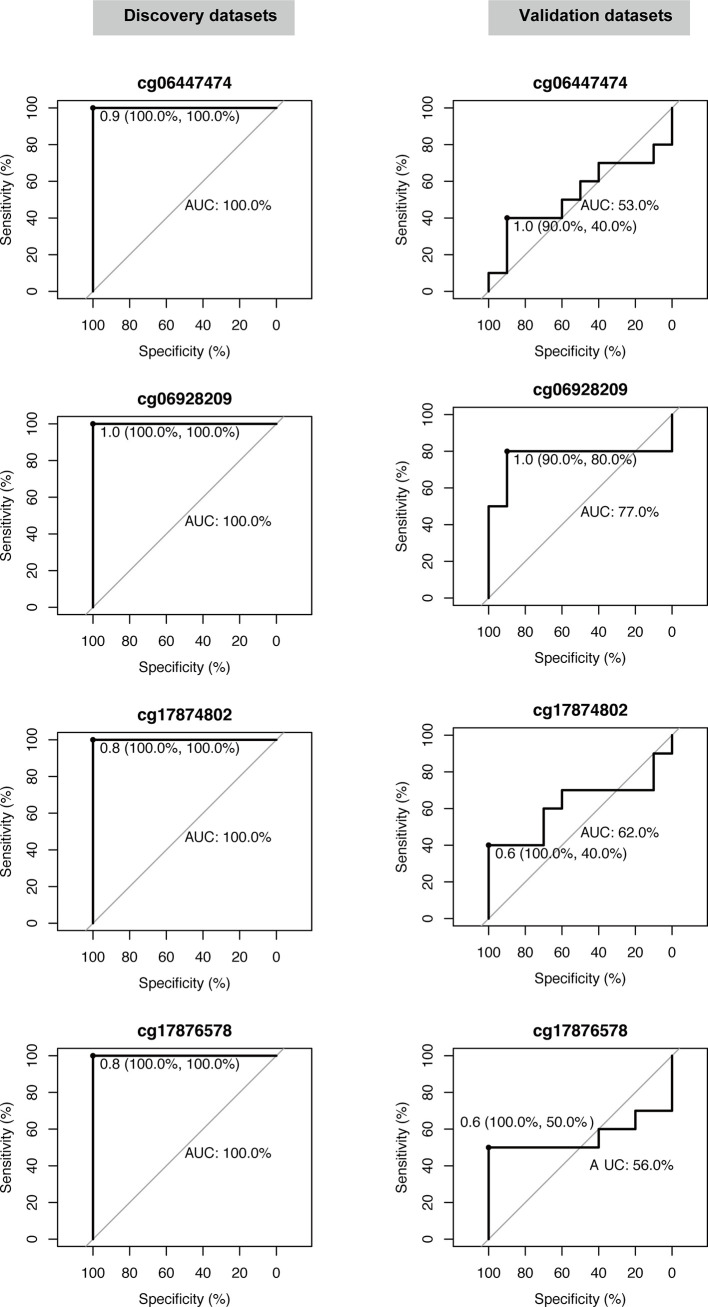
Validation of the diagnostic performance of four selected DNA methylation sites in the validation dataset. Receiver operating characteristic curve (ROC) analysis was conducted on this dataset. Area under the curve (AUC), specificity, and sensitivity are calculated and displayed for each dataset.

The five candidate methylation sites underwent pyrophosphoric acid sequencing to obtain the methylation level and only four of them were successfully evaluated. Then, ROC analysis was used to evaluate the diagnostic performance of each site on the discovery set and validation set respectively. As shown in **Figure 4**, all the four candidate sites were able to completely distinguish between the two sets of samples in the discovery set, with 100% sensitivity, specificity and AUC. In the validation set, the overall performance decreased, mainly due to the decrease in area under the curve, with the lowest AUC of only 0.53 (cg06447474); meanwhile, the specificity of each candidate site decreased, with the lowest specificity for cg06447474 and cg17874802, only 40%. However, the sensitivity of all candidate sites was high: cg06447474 90%, cg06928209 90%, cg17874802 100%, and cg17876578 100%. In summary, cg06928209 is the most promising of all candidate methylation sites as a molecular marker for early diagnosis, with 90% sensitivity, 80% specificity and AUC 0.77 on the external validation set.

## Discussion

The diagnosis of FTC before surgical resection remains challenging. Routine screening methods such as ultrasound or even FNAB could not diagnose FTC preoperatively. Accurate diagnosis may help to avoid unnecessary surgery in patient with benign thyroid lesions. Molecular tests were reported to be promising tools to aid pre-operative diagnosis of thyroid nodules, for example, a gene expression‐classifier based on 167 transcripts was tested in a multicentric study including 265 FNAB with indeterminate cytology (20). A high sensitivity (92%), but a low specificity (52%) was found in the identification of suspicious nodules. In this study, we analyzed DNA methylation and gene expression profiles in follicular thyroid cancer samples and benign thyroid lesion samples in our own cohort. One methylation site cg06928209 was identified as the potential biomarkers with high sensitivity and specificity in distinguishing FTC from BTL.

DNA methylation can effectively promote gene silencing and the hypermethylation status of CpG islands located in the promoter regions of tumor suppressor genes is thought to be the most common mechanism for inactivation of tumor suppressor genes in cancer development (21). Thus, detection of DNA methylation is thought to be a potentially effective screening means (22–28). RNA is a key component of gene transcription. However, from a biochemical point of view, both RNA and proteins are less stable than their DNA counterparts, which makes DNA-based markers particularly attractive, especially for non-invasive screening through blood and other body fluids (25–27). Yim JH. et al. performed differential DNA methylation analysis in 109 post-thyroid surgery tissue samples and a new diagnostic classifier for thyroid nodules was identified, with a sensitivity of 100% and a specificity of 97%. However, the study lacked external validation sets to further validate its performance and there were no FTC samples in their cohort (29). Mateus C. et al. used 70 thyroid cancer samples (mostly composed of PTC) and 17 BTL as training sets and the results showed a sensitivity of 90.7% and specificity of 75.4% for distinguishing between thyroid malignancies in postoperative tissue samples (30). Yet, their study was designed to identify potential biomarkers distinguishing malignancies and benign lesions rather than FTC and benign lesions specifically. In our study, we focused on the ability of molecular markers to distinguish FTC from BTL, with a sensitivity of 90.0% and a specificity of 80.0%.

As reported in previous studies, promoter hypermethylation is associated with transcriptional silencing of genes. Thus, opposing direction of gene expression and methylation status change was taken into consideration for the integrative analysis. This kind of integrated analysis was used in multiple studies in the past. For example, Zhiming L. et al. identified the cg23478805 methylation site on the ZCCHC13 gene as a marker for non-obstructive azoospermia (31) and Poirier JT. et al. analyzed a specific subgroup in small cell lung cancer (32). The role of the CXCL12 gene and its degree of methylation in the development of PTC was explored by Zhang S. et al. (33). However, as far as we know, very few studies used the same method to analyze FTC samples.

The limitation of this study is the relatively small training set due to the difficulty in FTC sample collection. This problem was also observed in other studies. For example, Mateus C. et al. included 70 thyroid cancer samples, but only 10 of them were FTC. Secondly, changing the proportion of samples in training set and validation set may help to increase the power of this study. More samples in the training set while less samples in the validation set should reduce the possibility of false positive, thus, 20 samples in training set and 10 samples in testing set may be a better design. In addition, only nodular goiter samples were selected to represent benign nodules in this study, because nodular goiter is the most common type in clinical practice and often coexists with other types of benign nodules. A more systematic study on the preoperative molecular diagnosis of FTC and follicular adenoma is planned for further research work.

In addition, we also investigated the performance of all combinations of selected methylation sites using logistic regression models and. However, the result (**Supplementary Table 2**) shows no significant improvement compared to the single site. It gives us a clue that maybe more sophisticated approaches are needed to extract additional information of significant differential methylated sites rather than simply combinations. For instance, the k-Nearest Neighbors, Support Vector Machine, Convolutional Neural Network and other machine learning approaches. Indeed, some studies has reported using machine learning approaches in analysis of multi omics, for example Zhang et al. used this comprehensive method to combine micro-RNA profile and protein sequencing to detect biomarkers for Grave’s disease and orbitopathy (34).

In summary, the differential methylation site cg06928209 was identified in this study by integrated analysis of RNA expression profile and DNA methylation profile, which shows high sensitivity and specificity in distinguishing FTC from BTL and can be used as a potential diagnostic tool.

## Conclusion

The four DNA methylation sites identified by this study, especially the cg06928209 site, can be used as a potential screening tool for detection and preoperative diagnosis of FTC patients. This assay has relatively high sensitivity and specificity, and is of great clinical value for the accurate diagnosis and treatment of thyroid nodules, especially for the identification of FTC and BTL by molecular means and the accurate preoperative diagnosis of FTC.

## Data Availability Statement

The datasets presented in this study can be found in the Gene Expression Omnibus (https://www.ncbi.nlm.nih.gov/geo/), accession number GSE197861.

## Author Contributions

The research project was designed by YY, WW, and ZC, then organized by XFW, LT, and XL. Samples were collected by YY and XMW. Laboratory work was conducted by TY and YW. Data analysis and the confirmation of selected sites was conducted by PX and ZM. The first draft of the manuscript was written by YY, and the manuscript was reviewed by LS, WW, LT, and XL. All authors contributed to the article and approved the submitted version.

## Funding

This study is supported by Grants from the National Natural Science Foundation of China (No. 81772853, 81972495, 81502786 and 81902719), Grants from National Natural Science Foundation of Zhejiang (No. LY18H160012, LQ18H120002 and LQ14H260003), and the Key Project of Scientific and Technological Innovation of Zhejiang Province (No. 2015C03031).

## Conflict of Interest

The authors declare that the research was conducted in the absence of any commercial or financial relationships that could be construed as a potential conflict of interest.

## Publisher’s Note

All claims expressed in this article are solely those of the authors and do not necessarily represent those of their affiliated organizations, or those of the publisher, the editors and the reviewers. Any product that may be evaluated in this article, or claim that may be made by its manufacturer, is not guaranteed or endorsed by the publisher.
