# Supplementary material for: Integrative Analysis of DNA Methylation and Gene Expression Identified Follicular Thyroid Cancer-Specific Diagnostic Biomarkers
Source: Front Endocrinol (Lausanne). 2022 Mar 14;12:736068. doi: 10.3389/fendo.2021.736068 (PMC8964406; doi:10.3389/fendo.2021.736068)
Supplement: Supplementary file 1 [file Table_1.docx]

Supplementary Material

# Supplementary Figures and Tables

## Supplementary Table

| **Table 1 Clinical characteristic of study cohort** | | | | | | | |
| --- | --- | --- | --- | --- | --- | --- | --- |
| Characteristic | | Total cohort (n = 30) | | Training cohort (n = 10) | | Testing cohort (n = 20) | |
|  |  | Group | | Group | | Group |  |
|  |  | FTC (n = 14) | BTL (n = 16) | FTC (n = 4) | BTL (n = 6) | FTC (n = 10) | BTL (n = 10) |
| Gender , No. (%) | Male | 7 (50%) | 5 (31%) | 3 (40%) | 3 (50%) | 4 (40%) | 2 (20%) |
|  | Female | 7 (50%) | 11 (69%) | 1 (60%) | 3 (50%) | 6 (60%) | 8 (80%) |
| Age, mean ± SD | | 39.57 ± 18.06 | 54.44 ± 10.46 | 39.75 ± 16.46 | 52.67 ± 12.47 | 39.50 ± 19.52 | 55.50 ± 9.63 |
| Tumor diameter(cm), mean ± SD | | 3.41 ± 1.29 | 4.17 ± 1.04 | 3.25 ± 0.19 | 4.42 ± 0.38 | 3.47 ± 1.55 | 4.02 ± 1.29 |
| T , No. (%) | T1a | 1 (7%) | / | 0 (0%) | / | 1 (10%) | / |
|  | T2 | 10 (71%) | / | 4 (100%) | / | 6 (60%) | / |
|  | T3a | 3 (22%) | / | 0 (0%) | / | 3 (30%) | / |
| N , No. (%) | N0 | 14 (0%) | / | 4 (100%) | / | 10 (100%) | / |
| M , No. (%) | M0 | 14 (100%) | / | 4 (100%) | / | 10 (100%) | / |
| Stage , No. (%) | I | 12 (86%) | / | 4 (100%) | / | 8 (80%) | / |
|  | II | 2 (14%) | / | 0 (20%) | / | 2 (20%) | / |

**Supplementary Table 1.** Clinical information of the total cohort, training cohort and testing cohort.

| **Methylation sites Combination** | **AUC** | **Sensitivity** | **Specificity** |
| --- | --- | --- | --- |
| cg06447474 + cg06928209 | 79 | 0.7 | 0.9 |
| cg06447474 + cg17874802 | 63 | 0.3 | 0.9 |
| cg06447474 + cg17876578 | 58 | 0.2 | 1 |
| cg06928209 + cg17874802 | 78.5 | 0.8 | 0.8 |
| cg06928209 + cg17876578 | 81 | 0.7 | 0.9 |
| cg17874802 + cg17876578 | 71.5 | 0.6 | 0.9 |
| cg06447474 + cg06928209 + cg17874802 | 79 | 0.7 | 0.9 |
| cg06447474 + cg06928209 + cg17876578 | 81 | 0.7 | 0.9 |
| cg06447474 + cg17874802 + cg17876578 | 73 | 0.3 | 0.9 |
| cg06928209 + cg17874802 + cg17876578 | 79 | 0.7 | 0.9 |
| cg06447474 + cg06928209 + cg17874802 + cg17876578 | 78 | 0.7 | 0.9 |

**Supplementary Table 2.** Classifiers trained by logistic regression using selected methylation sites and their different combinations were not superior than simply using a single methylation site.
